# Surgical Interference in Rectal Disorders

**Published:** 1895-12

**Authors:** J. McFadden Gaston

**Affiliations:** Atlanta, Ga.


ATLANTA

Medical and Surgical Journal.

VOL. XII. DECEMBER, 1895. No. 10.

LUTHER B. GRANDY, M.D., MANAGING EDITOR.

M. B. HUTCHINS, M.D., BUSINESS MANAGER.

COLLABORATORS:
H. V. M. MILLER, M.D., LL.D., VIRGIL O. HARDON, M.D., FLOYD W. McRAE, M.D., DUNBAR ROY, M.D., AND H. R. SLACK, M.D., PH.M.

### *ORIGINAL COMMUNICATIONS.*

### SURGICAL INTERFERENCE IN RECTAL DISORDERS.*

BY J. McFADDEN GASTON, M.D.,

ATLANTA, GA.

The rectum is an ovoidal canal extending from the sigmoid flexure of the colon to the anus. It is narrowest above and below, presenting a pouch-like shape, capable of great expansion, and serving as a reservoir for the débris, which results from the completion of the process of alimentation. Its walls are thicker than those of the colon and capable of great contraction in expelling the excrement. The serous coat invests its upper part and forms the meso-rectum and extends on the sides sparingly, but more in front. The rectal fascia, of fibro-connective tissue, with its blood-vessels, surrounds the lower portion of the rectum.

" There are two sets of muscular fibers encircling the lower part of the rectum, known as the outer and inner sphincters, by which

*Read before the Southern Surgical and Gynecological Association, at the meeting in Washington City, November 12, 1895.

it is automatically closed except when dilated voluntarily for the release of flatus or the discharge of fecal matter. There is also another distribution of circular contractile muscular fibers at the upper limit of the rectum, which constitutes the division between it and the sigmoid flexure of the colon. This may be appropriately designated as the recto-colic sphincter, and forms an effective barrier ordinarily to the descent of the excrement into the rectum. This annular muscle has not received from anatomists or physiologists the consideration which its rôle in the intestinal functions warrants. It is a veritable constriction from muscular contraction, by which the colon is normally closed against the descent of the fecal mass into the rectum." Before the excrementitious matter reaches this constrictor it is deprived of its nutriment, and after passing this annular division, it is deposited in the reservoir below until a convenient opportunity is afforded for its expulsion through the anal outlet.

The multiform departures of the tissues of the rectum from their normal condition induces various pathological modifications and functional derangements. There are not only changes of structure from ordinary inflammatory processes, but also material alterations in the constituent elements of the organism from a deterioration of vitality induced by syphilitic and tuberculous degeneration, as well as a profound depravity of structure from the development of carcinomatous neoplasms.

Simple fissure of the anus, involving the mucous and submucous tissues of the rectum, may prove a source of reflex disturbance to the nervous system, so as to require operative measures for relief. Hemorrhoidal developments, whether confined within or protruding from the anal opening, may be complicated with sanguineous exudation, demanding a surgical procedure. Strictures of the rectum from fibrinous depositions in its walls call for division or excision of the structures involved.

Fistula in ano, resulting from perirectal abscess, may be complete or incomplete, and admits of no radical cure without operation, even when associated with tubercular disease. Papillomatous nodules, located in the walls of the rectum, whether limited to a small area or extending over a considerable portion of the mucous membrane, should be removed, with a view to obviate their degeneration into a malignant form of disease. When carcinomatous induration of the rectal tissues is detected early, there is encouragement to undertake an operation, but after the breaking down of the neoplasm with infiltration of surrounding structures, no benefit is derived from excision of the parts involved.

It is a mooted point in regard to the practicability of eradicating rectal troubles of syphilitic origin by medication, and with the present light on the subject it seems justifiable to resort to such a surgical measure as the condition indicates, while constitutional treatment is being carried out in the case.

There are instances of supposed development of specific diseases in the form of stricture of the rectum, after the lapse of many years subsequent to any syphilitic contamination, and some authors claim their ability to diagnosticate specific stricture even without a previous history of primary syphilis. But the medical profession should be very guarded in coming to such a conclusion upon a general consideration of the nature of the case; and without sufficient evidence of the initial lesion in a patient of respectable position in society, no theoretic bias should lead to a diagnosis of syphilis.

There is a rectal relaxation consisting in prolapse of the intestinal wall, which, in young subjects, is often relieved without resorting to any operative measure. But again this occurs in adults or even in old subjects, when nothing short of active surgical interference can be relied upon for relief, and even with the most vigorous measures, prolapse of the rectum proves, in some cases, intractable.

There is not infrequently an ulceration extending around and within the lower part of the rectum, involving the glandular structure near the anal outlet, which is only amenable to excision. This has been mistaken for the breaking down of hemorrhoids, but is quite distinct in its pathological condition, and should not be confounded with other ulcerative degeneration of the rectal tissues.

While there are some rectal disorders besides those enumerated which call for the surgeon's attention, it is not requisite to enter into their consideration in this general summary of the conditions demanding operative interference.

It will be perceived that the rectum affords material for surgical work of the most important character, and it should not be relegated to those professing to deal with so-called orificial surgery.

The readers of the *Annual of the Universal Medical Sciences*, for 1895, will note that Dr. C. B. Kelsey limits his contribution to syphilitic and cancerous disease of the rectum. I have noticed, however, that another specialist in this department, Dr. Joseph M. Mathews, is arousing the dormant energies of the profession to the various phases of rectal troubles through his publications, and especially in his *Quarterly Journal.* While the operation of Kraske and others for the extirpation of the rectum does not receive his sanction, yet there are desperate cases in which some form of removal is warranted, rather than leave a patient to die without any operation.

This matter of rectal surgery is not viewed from the standpoint of the specialist, as my attention has not been directed to it except as a part of my work in the rather extended field of a general surgeon. But I am fully impressed with the conviction that many cases find their way into the hands of quacks which ought to be treated by members of the regular medical profession, and preferably by those who have made a special study of rectal diseases and are prepared to treat properly all the surgical disorders of the rectum. In the paper by Dr. Arpad G. Gerster, before the American Surgical Association, upon the surgery of the rectum, in 1893, there is a full résumé of cases treated during four years in the Mount Sinai Hospital of New York. There were 557 patients suffering from rectal ailments admitted within this period; of these 280 were classed as hemorrhoids; 167 were cases of fistula, including the more acute forms of ischio-rectal trouble; 17 cases of carcinoma; 11 cases of prolapse; 6 case of cicatricial stricture; 6 cases of chronic ulcers; 7 cases of polypus; 1 case of multiple adenoma; 2 cases of congenital atresia of the anus and one of the rectum, with 4 cases of anal fissure.

It will be observed that hemorrhoids and fistulæ make up four-fifths of the entire number of cases involving the rectum, and it seems remarkable that only four cases of fissure should have appeared, making an average of one to each year, when this is generally regarded as of frequent occurrence. Dr. Gerster performed extirpation of the rectum in five cases, four times for carcinoma, and once for strictures caused by ulcerative proctitis. Two of these died of acute anemia in consequence of the operation; one suffering from carcinoma, the other from ulcerative proctitis. In both, the operation of Kraske was performed. Two other cases, in which about six inches of the gut were removed by Kraske's operation, were successful. "In the case of a woman fifty years old, whose very wide pelvic aperture permitted easy access without extirpation of the sacrum, the coccyx alone being excised, four and a half inches of the rectum were removed according to the old-fashioned perineal method. She made an easy and rapid recovery." By Gerster a preference is given to the radical operation over colotomy, where the condition of the patient permits it, but the patients should be carefully selected, in view of the general powers of resistance in the patient rather than in the extent of the local disease which is encountered. It is urged that only such cases should be selected as have a good circulation and whose heart and blood supply are fairly preserved. Preliminary colotomy is enjoined where much fecal distress and more or less fever exist; while preparatory feeding and general regimen are held to be essential for success.

In the discussion of this paper, Dr. Lewis S. Pilcher, of Brooklyn, presents some interesting data from the service of Dr. Fowler and himself in the Methodist Episcopal Hospital of Brooklyn, N. Y., relative to carcinoma of the rectum. Of ten cases under observation in the last five years, three presented such an extent of local disease and general cachexia that operative interference by extirpation was inadvisable. In the fourth case, in which the disease extended four inches above the anus, operation was refused. In the other six cases, operations were done with two deaths as the immediate result, and without a radical cure in either of the other four cases, though life was prolonged for some time. One of these patients was twenty-nine years old, and another only twenty-three years.

Dr. H. H. Mudd, of St. Louis, states that he has removed the upper portion of the rectum and the lower portion of the colon through the abdominal cavity with success.

Dr. L. McLane Tiffany, of Baltimore, says that by proctotomy he has utterly failed to give relief in rectal troubles, and he believes that colotomy gives the best results.

Dr. T. F. Prewitt, of St. Louis, claims that a great many cases of cancer of the rectum are not suitable for excision, and that inguinal colotomy is the proper course in such cases. He thinks it is better to make a complete section of the colon and bring both ends of the bowel out. In this way you avoid the passage of any matter through the lower part and do not need any spur.

In closing the discussion, Dr. Gerster remarked that he had not mentioned closure of the colon after extirpation of the rectum, but he had reclosed the opening into the colon when the cause for which the colotomy was done had been removed. His section being transverse, including almost the entire circumference of the bowel, both apertures protrude through the external wound. He believes that the spur is essential in colotomy.

In an editorial review of operative measures for cancerous affection of the rectum in the *Annals of Surgery*, by Dr. J. P. Warbasse, a full report of those having large experience in the treatment of this class of cases is presented. I would refer those interested in deciding upon the practicability of affording relief in carcinoma of the rectum, to this concise and impartial record without entering into minute details of the observations. It may be stated, however, in general terms, that conflicting views are presented as to the stage at which operative measures of relief are admissible, and no definite conclusion can be arrived at in regard to the precise steps which are indicated, or as to the mode of proceeding in different cases. An operation adapted only to women is detailed with some confidence in its advantage, which, so far as my observation goes, has not been practiced in this country. It consists in dividing the whole perineal body into the rectum, and thus secure an ample field of operation, which is subsequently to be reunited by sutures.

The burning and urgent appeal to the surgeon to-day is for a definite settlement of the issue as to active interference in cases of pronounced cancer of the rectum. Shall we content ourselves with the mere palliative measures of inguinal colotomy and leave the diseased structures untouched, as urged by Dr. Matthews, in his paper before the American Medical Association; or shall we endeavor to remove all the tissues involved by extirpation, as recommended by Dr. Gerster, in his paper presented to the American Surgical Association? The full statistics of results in the hands of skilled operators ought to be collected and a fair analysis made before a final adjudication of the question can be reached. The materials for such a comparison should be obtained from cancer hospitals in this country and other countries, as well as from general hospitals receiving and treating this class of patients, and being grouped together, a fair inference may be drawn as to the feasibility of active interference in any case of carcinoma of the rectum.

